# Effects of Different Vegetable Rotations on Fungal Community Structure in Continuous Tomato Cropping Matrix in Greenhouse

**DOI:** 10.3389/fmicb.2020.00829

**Published:** 2020-05-20

**Authors:** Jian Lyu, Li Jin, Ning Jin, Jianming Xie, Xuemei Xiao, Linli Hu, Zhongqi Tang, Yue Wu, Lijuan Niu, Jihua Yu

**Affiliations:** College of Horticulture, Gansu Agricultural University, Lanzhou, China

**Keywords:** tomato, continuous tomato cropping, crop rotation, organic ecotype soilless culture substrates, fungal community

## Abstract

Intensive greenhouse vegetable cultivation aggravates continuous cropping, resulting in the disturbance of the microbial community structure and the diversity of the soil matrix. In this study, we evaluated the diversity of the rhizosphere matrix fungi in rotation and continuous cropping systems by using high-throughput sequencing analysis of substrates under 6-years of continuous tomato cropping and rotation with cabbage, bean, or celery in greenhouse pots. The results showed that fungal richness in the Chinese cabbage rotation treatment (B) was significantly lower than that of other treatments, and fungal diversities of treatment B and the bean rotation treatment (D) were significantly lower than that of continuous tomato cropping (CK). Contrastingly, the celery rotation treatment (Q) increased the fungal diversity and richness. Furthermore, a principal coordinate analysis showed that the fungal soil community structure of each rotation treatment was different from that of CK. The relative abundances of several harmful fungi (such as *Pseudogymnoascus*, *Gibberella*, and *Pyrenochaeta*) in control CK were significantly higher than those in rotation treatments. In addition, the matrix electrical conductivity, organic matter, total K, and available P in treatments B and D were significantly higher than those in control CK. Moreover, pH and total N of treatment Q were significantly higher than those of control CK. Most fungi were positively correlated with organic matter and available P and negatively correlated with pH. Therefore, rotation with celery could improve the abundance and diversity of fungi in continuous tomato cropping substrates and reduce the relative abundance of harmful fungi. These results indicated that the rotation of celery and tomato could effectively maintain the ecological balance of the substrate microenvironment and provide a more effective way to prevent the problems of continuous tomato cropping in greenhouse.

## Introduction

Continuous cultivation of greenhouse tomatoes lead to soil acidification and salinization, which seriously restricts the sustainable development of vegetable production in sunlight greenhouses (Liu et al., 2014). As a new method of vegetable crop cultivation, nutrient cultivation in a matrix has been gradually developed worldwide (Liu et al., 2009; Maboko et al., 2013). However, the nutrient matrix might be affected after years of continuous cultivation, and substrates need to be replaced continuously, which would cause a large consumption of human and material resources. For example, Van Assche and Vangheel (1993) found that the substrate of continuous cultivation was easily produced pathogenic bacteria, which caused plant diseases. However, continuous cropping of a single crop often leads to an imbalance in soil microbial communities, which is manifested in the decrease of microbial diversity, increase of pathogenic bacteria, and decrease of beneficial bacteria (Lu et al., 2013; Li et al., 2014; Fu et al., 2017). For instance, several studies have reported that continuous cropping reduced many microbial species and organic matter in the soil, leading to the occurrence of soil-borne diseases (Brussaard et al., 2007; Liang et al., 2011; Han et al., 2016). Soil-borne diseases of plants are a perennial epidemic, usually causing diseases of plant roots and sometimes endangering the whole plant, and these diseases are easily affected by the soil environment and cultivation measures. The pathogens of soil-borne diseases presented in plants include fungi, bacteria, actinomycetes, and nematodes, among which fungi are often dominant; for example, *Fusarium oxysporum* caused Fusarium wilt in Solanaceae. Moreover, *Verticillium* has been demonstrated to be the agent of vascular wilt, and *Pyrenochaetalycopersici* is considered as the vector of root rot. However, little is known about the effects of continuous cropping on microorganisms in the substrate. Therefore, it is of great significance to study the changes of the fungal community in the nutrient matrix after rotation of different vegetables in order to explore the ways to prevent and control continuous cropping obstacles in tomatoes.

In continuous cropping systems, crop rotation can effectively increase crop yields and reduce the incidence and severity of soil-related factors (Tian et al., 2011; Wright et al., 2015). Studies have shown that crop rotation can increase the absorption of nutrients, increase crop yield, and significantly increase the content of organic carbon, total nitrogen, and the total microbial population in the soil (Tian et al., 2009; Tao et al., 2015; Jahan et al., 2016; Lee et al., 2016). To improve soil microbial diversity by rotation may require the use of specific crop combinations, which are expected to have a greater impact on soil microbial diversity. For example, in the substrate of Chinese cabbage–tomato rotations, some studies have found that total microbial biomass and bacterial count increased significantly compared to those of winter fallow substrate, while the number of fungi decreased, and the proportion of fungi to bacteria also decreased (Wei et al., 2012). Moreover, intercropping leek with celery has been reported to have many beneficial effects, such as reducing weeds and pests without hindering cultivation (Baumann et al., 2000, 2001). Additionally, the occurrence of bacterial wilt in tomatoes was largely controlled during tomato–cowpea intercropping (Jing, 1999).

At present, studies on the soil microbial community in continuous cropping systems have mainly focused on bacteria (Zhang et al., 2010; Jun et al., 2014; Zhao et al., 2014), and a large number of bacterial populations have been shown to contribute to antimicrobial disease prevention, plant growth, or systemically induced plant resistance (Garbeva et al., 2004; Weller et al., 2006; Rodrigo et al., 2011). However, studies also have shown that fungi, as one of the key components of microbial populations, could can affect the stability and fertility of soil structure and could also have a wide range of key ecological functions, including decomposition, parasitism, pathogenesis, and symbiosis (Penton et al., 2014). In China’s intensive vegetable production system, the effects of long-term continuous cropping on soil quality, especially on genetic and functional diversity of fungi, have not been widely acknowledged in cultivation. As we all know, high-throughput sequencing is a powerful tool for the study of microbial communities and has been widely used in many studies (Tago et al., 2014; Sommermann et al., 2018).

Therefore, the purpose of this study was to use high-throughput sequencing method to study the change trend of fungal diversity and community structure in a continuous cropping substrate after rotation of different vegetables and tomato substrates that had been planted for 6 years and 12 crops. We made two hypotheses: (1) the long-term continuous cropping of tomato would increase the abundance of harmful fungi in the substrate, while the implementation of rotation of different vegetable/tomato would lead to the change of fungal diversity, and the change of fungal community in the substrate is closely related to the physical and chemical properties of the substrate because most of the rhizosphere microorganisms are based on carbon sources in the substrate; and (2) different types of rotation vegetables have different effects on the substrate fungal community, different vegetable roots have different abilities to absorb nutrients, some vegetables contain strong allelochemicals, and the response of the root fungal community to them will also be different. To address these points, we compared the composition of fungal community in the substrate of different vegetables (cabbage, bean, and celery) rotation and tomato continuous cropping, and we analyzed the correlation between the two in combination with the physical and chemical properties of the substrate. Finally, we evaluated which vegetable rotation is better for quality tomato cultivation.

## Materials and Methods

### Experiment Design

The test substrate was an organic ecotype soilless culture substrate (the substrate formula was the mixture of slag: spent mushroom: cow manure: chicken manure: corn straw = 13:5:5:2:14). From June 2012 to June 2018, the continuous cropping experiment was conducted in the solar greenhouse of “Zongzhai non-cultivated land facility agricultural demonstration park” in Zongzhai Town, Suzhou District, Jiuquan City, Gansu Province, China (98° 20′ ∼ 99° 18′ E, 39° 10′ ∼ 39° 59′N). It is a typical continental climate. The average sea level is 1360 m, and the annual average temperature is 7.3°C with an annual average precipitation of 176 mm and sees annual sunshine hours of 3033–3316 h. The continuous cropping vegetable is tomato (*Lycopersicon Esculentum* Mill.), which was planted twice a year. The overwintering crop is generally raised in September of that year, planted in October, collected in the first 10 days of February of the next year, and pulled in time in May. Summer and autumn crops are generally planted in time according to the overwintering time. Most of them are planted in June, they are listed in the first 10 days of August, and they are planted in time in October. The experimental tomato variety was “Jingfan 501,” a pink fruit of infinite growth type, with a plant spacing of 45 cm, row spacing of 25 cm, and 30 plants in each plot. After continuous cropping, the substrates contained total K: 11.78 (g⋅kg^–1^), total P: 1.31 (g⋅kg^–1^), total N: 0.51 (g⋅kg^–1^), available P: 82.81 (mg⋅kg^–1^), available K: 63.17 (mg⋅kg^–1^), alkali-hydrolyzable N: 907.67 (mg⋅kg^–1^), EC: 1683.67 (μS⋅cm^–1^), and pH: 6.37.

The rotation experiment was conducted in the glass greenhouse of Gansu Agricultural University from August 2018 to March 2019. The rotation vegetables included cabbage (*Brassica pekinensis* Rupr.), bean (*Phaseolus vulgaris* Linn.), and celery (*Apium graveolens* L.). The continuous cropping substrates collected in the continuous cropping experimental site was transported to the greenhouse of Gansu Agricultural University and then put into a 19 cm × 30 cm pot. The amount of matrix in each pot was 5 kg. The seedlings that had been raised in advance were moved into the basin, and the field management measures of each treatment were consistent with the local conventional management measures.

In the rotation experiment, there were three treatments: cabbage rotation (treatment B), bean rotation (treatment D), and celery rotation (treatment Q). The control was the continuous cropping tomato (control = CK), and each treatment had three repetitions.

### Matrix Sampling

The substrate samples were collected after rotation plant seedling pulling (March 2019), and 12 samples were selected for each treatment. After removing the 0–5 cm surface matrix and gently shaking off the matrix around the root system, the matrix adhered to the root surface was brushed off for collection and immediately stored as rhizosphere in an ice box. Subsequently, these samples were divided into two parts, one part was air-dried to determine the physical and chemical properties of the substrate, and the other part was used to extract substrate microbial DNA. There were four treatments in this experiment, and each treatment took three samples for DNA extraction, leaving a total of 12 samples.

### Determination of Matrix Physicochemical Properties

Physical and chemical properties of the substrate were determined following Bao’s (2000) method. The pH value of the substrate water suspension (1:5) was determined by glass electrode (PHS-3E; ShanghaiJingke, China). The conductivity of the substrate was mixed in the ratio of the substrate (water = 1:5), placed on the oscillator, vibrated for 30 min, and then filtered. The readings are measured by inserting DSJ-308A conductivity meter of Shanghai Jingke (DSJ-308A, ShanghaiJingke, China) into the filtrate. The content of organic matter in the matrix was assessed by the potassium dichromate method. Alkali-soluble N, available P, and available K were determined by the alkali diffusion method, molybdenum blue colorimetric method, and flame photometry, respectively. Total N, total P, and total K were determined by the Kjeldahl method, molybdenum antimony colorimetric method, and flame spectrophotometer (FP6410, Shanghai, China), respectively.

### DNA Extraction

Total DNA of substrate microorganisms was extracted from 0.5 g mixed soil samples using an EZNA^®^ Soil DNA Kit (OMEGA, Bio-Tek, Norcross, GA, United States) according to the manufacturer’s protocols. Each composite substrate sample was extracted in triplicate, and the extracted DNA solutions were pooled.

### Quantitative PCR and Illumina MiSeq Sequencing

The purified DNA was used as a template, and the fungal ITS2 region PCR amplification was carried out using primers fITS7 (5′-GTGARTCATCGAATCTTTG-3′) and ITS4 (5′-TCCTCCGCTTATTGATATGC-3′) (Bokulich et al., 2013). The 25 μL reaction system consisted of 12.5 μL Phusion Hot Start Flex 2 × Master Mix, 2.5 μL forward primer, 2.5 μL reverse primer, 50 ng template DNA, and dd H_2_O added up to volume. The PCR reaction was carried out on an ABI GeneAmp^®^ Model 9700 (Applied Biosystems, Foster City, CA, United States). The amplification conditions involved pre-denaturation at 98°C for 30 s, denaturation at 98°C for 10 s, annealing at 54°C for 30 s, extension at 72°C for 45 s, for a total of 35 cycles, and a final extension at 72°C for 10 min. Each sample was prepared in three replicates and the final PCR amplification product was detected by 1% agarose gel electrophoresis and purified by using an AxyPrep DNA Gel Extraction Kit (AxyGenBioScIsStates, Union City, CA, United States) using quantitative fluorescence. The purified PCR products were quantified by Quant-iT PicoGreen dsDNA Assay Kit on Promega QuantiFluor fluorescence quantitative system. The qualified library concentration should be more than 2 nM. After diluting the qualified on-line sequencing libraries (the index sequence is not repeatable), they were mixed according to the required sequencing amount according to the corresponding proportion, and transformed into a single chain by NaOH for on-line sequencing; using the MiSeq sequencer for 2 × 250 bp double terminal sequencing, the corresponding reagent is MiSeq Reagent Kit v2500 cycles.

### Processing of Sequenced Data

The original data obtained by sequencing was an image file. After base calling, the resulting file was saved in a fastq format. QIIME (version 1.17; Caporaso et al., 2010) was used to filter the quality of the fastq file. PEAR (version 0.9.6; Zhang et al., 2014) and Vsearch (version 2.3.4; Rognes et al., 2016) software were used to splice the two terminal sequences of the original data and filter the chimeric sequence. By using a Vsearch (version 2.3.4; Rognes et al., 2016) algorithm, the sequences with a similarity greater than 97% were clustered, and then the OTU representative sequences obtained by clustering analysis were compared with the RDP database (version 11.5; Cole et al., 2009, 2014) and UNITE database (version 7.2; Nilsson et al., 2019) to get the species annotation results of all OTUs.

### Statistical Analysis

Statistical analyses of data were performed using the R packages Stats and Vegan (version 2.3-5; Oksanen et al., 2016). Alpha-diversity indices (Shannon index, Simpson index, Chao1 index, and a number of observed species) were calculated using QIIME (alpha_diversity.py). For β-diversity analysis, a cluster analysis (sample clustering by Bray-Curtis distance) was used to show the similarity between samples, and weighted UniFrac distance measurement (based on system development structure) is used to generate PCoA map to further evaluate the similarity between community members of the samples (Lozupone et al., 2006). According to the sample species abundance table, a Kruskal Wallis non-parametric test and Dunet t test were used to judge whether there was significant difference between different groups, and multiple comparison corrections were done with Benjamini-Hochberg FDR. Generally, *P* < 0.05 was considered as the significant difference (R Core Team, 2017). A redundancy analysis (RDA) was performed by the RDA function in the “vegan” package in “R” (version 2.1.3), which was used to study the effect of physical and chemical properties of rhizosphere matrix on the composition of rhizosphere matrix fungal community.

One-way analysis of variance (ANOVA) and Least Significant Difference (LSD) were applied to evaluate the effects of different tillage methods on physical and chemical properties of substrate.

## Results

### Effects of Rotating Different Vegetables on Physicochemical Properties of Tomato Continuous Cropping Substrate

After the rotation of different vegetables, the substrate pH and alkaline-N of treatments B and Q were significantly higher than those of control CK (Table 1). Compared to those of control CK, substrate pH and alkaline-N increased by 1.63 and 2.93%, respectively, in treatment B and by 4.59 and 5.6%, respectively, in treatment Q. The EC, organic matter, total K, and available P of the B and D treatment were significantly higher than those of control CK. In treatment B, EC, organic matter, total K, and available P increased by 31.2, 7.4, 10.99, 13.65%, respectively, while in treatment D they increased by 3.38, 16.75, 26.42, and 8.91%, respectively, compared with the control (Table 1). In treatment Q, EC, total P, total K, and available K were significantly lower than those in treatment F, by 55.38, 20.63, 13.69, and 61.38%, respectively.

**TABLE 1:** Physicochemical properties of substrates treated with different treatments.

**Treatments**	**pH**	**EC**	**Organic matter**	**Total N**	**Total P**	**Total K**	**Available P**	**Available K**	**Alkaline N**
		**(μS⋅cm^–1^)**	**(g⋅kg^–1^)**	**(g⋅kg^–1^)**	**(g⋅kg^–1^)**	**(g⋅kg^–1^)**	**(mg⋅kg^–1^)**	**(mg⋅kg^–1^)**	**(mg⋅kg^–1^)**
B	6.870.04b	1215.336.06a	104.492.19b	1.831.06b	1.30.01a	12.730.23b	73.950.72a	35.50.46c	858.085.34b
D	6.730.06c	957.674.41b	113.592.19a	2.570.23b	1.320.05a	14.50.15a	70.870.14b	46.830.12a	861.472.44b
Q	7.070.01a	413.332.03d	94.441.83c	2.330.23b	10.01b	9.90.06d	66.450.48c	14.470.15d	880.255.05a
CK	6.760.01c	926.331.45c	97.292.81c	6.230.46a	1.260.06a	11.470.12c	65.071.65c	37.470.49b	833.587.45c

**FIGURE 1:**
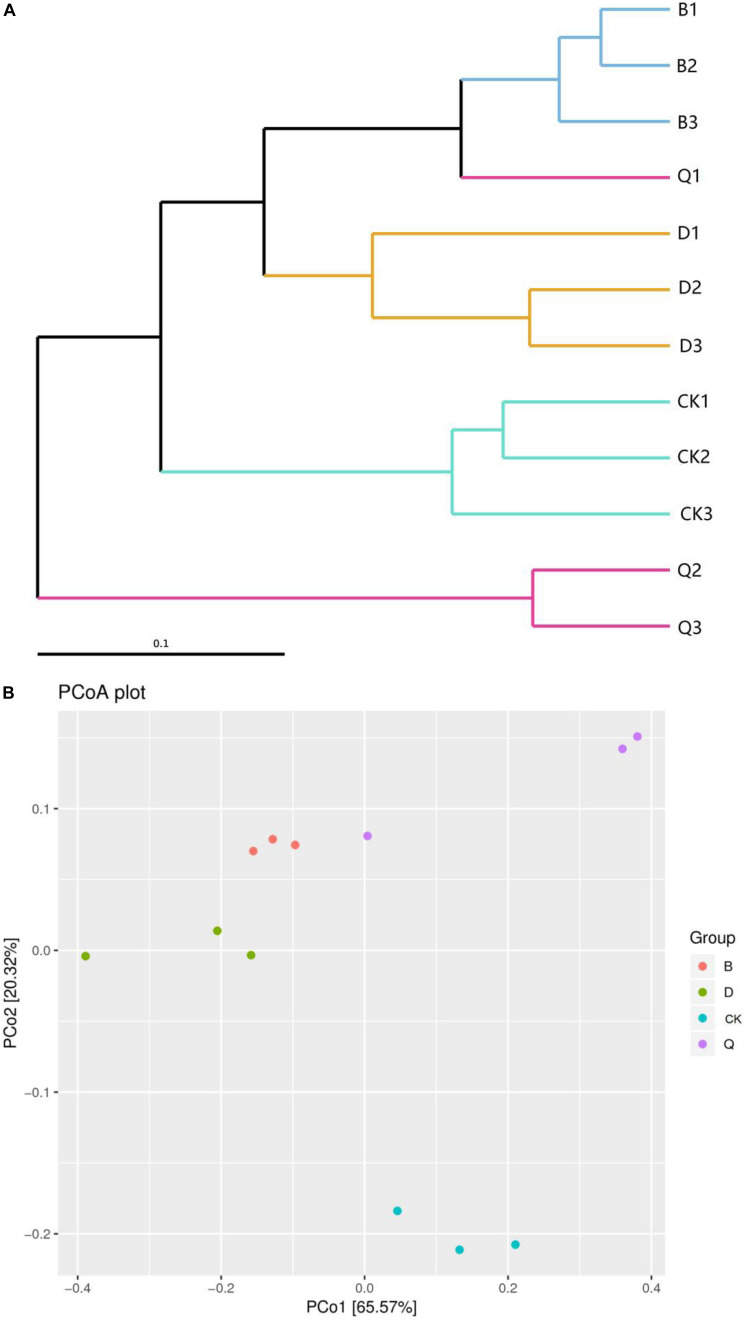
Hierarchical clustering **(A)** and principal coordinates analysis **(B)** of fungal communities in tomato cropping substrate based on different treatments. Treatments B, D, and Q refer to crop rotation with Chinese cabbage, kidney bean, and celery, respectively. Control CK is continuous cropping with tomato.

*EC, electrical conductivity; PH, acidity, and alkalinity; B, D, Q, and CK are defined in Figure 1. Treatments B, D, and Q refer to crop rotation with Chinese cabbage, kidney bean, and celery, respectively. Control CK is continuous cropping with tomato.*

### Effects of Different Vegetable Rotations on the Diversity of Fungi in Tomato Continuous Cropping Substrate

#### Fungal α-Diversity

Substrate sample abundance (observed species and Chao1 index) and diversity (Shannon and Simpson indices) are shown in Table 2. The Chao1 index and observed species mainly reflect the number of OTUs in the sample. Treatment B had the lowest observed species and Chao1 index, while treatment Q had the highest values. These results indicate that the fungi in treatment B had the lowest abundance. Fungi in treatments Q and control CK had the highest and second highest abundances, respectively. Shannon and Simpson indices also reflect the number of species in the sample and the average or uniformity of species abundance in the sample. Treatments B and D had significantly lower Shannon and Simpson indices compared with control CK (Table 2).

**TABLE 2:** The richness and diversity index of fungi community in different rotation vegetables and continuous tomato were analyzed and observed.

**Treatments**	**Observed species**	**Shannon**	**Simpson**	**Chao1**
B	200.3321.93c	2.950.42c	0.640.02b	281.8726.46b
D	267.677.64b	3.470.1b	0.700.04b	296.5562.93ab
Q	29521.93a	4.650.25a	0.950.02a	366.7421.58a
CK	282.336.81ab	5.060.09a	0.900.02a	332.8534.85ab

*Values are presented as means ± standard deviation (n = 3). Different letters indicate statistically significant differences (P < 0.05). B, D, Q, and CK are defined in Figure 1.*

#### Fungal β-Diversity

Hierarchical clustering was used to analyze β-diversity of fungal communities in the continuous tomato cropping system (Figure 1A). The hierarchical clustering resolved the fungal community into four clusters, one composed of group B, one of group D, one of group Q, and one of CK. Groups B and D were clustered together and separate from the control CK. In addition, fungal community from treatment Q differed the most from those in treatments B, D, and control CK.

The Unifrac weighted PCoA based on OTUs also clearly showed the differences among different vegetable rotations and continuous tomato cropping samples. PCo1 and PCo2 explained 65.57 and 20.32% of the total variability of fungal data, respectively. The fungal community in the sample series of continuous tomato cropping (CK) was clearly separated from the other nine samples by PCo2. The fungal community members of the cabbage (B) and the bean (D) rotation matrix samples were the most similar (Figure 1B). The unweighted unifrac algorithm showed similar results, but, for the sake of clarity, only the weighted Unifrac-PCoA plot is shown here.

### Effects of Different Vegetable Rotations on Fungal Community Composition in Tomato Continuous Cropping Substrate

**FIGURE 2:**
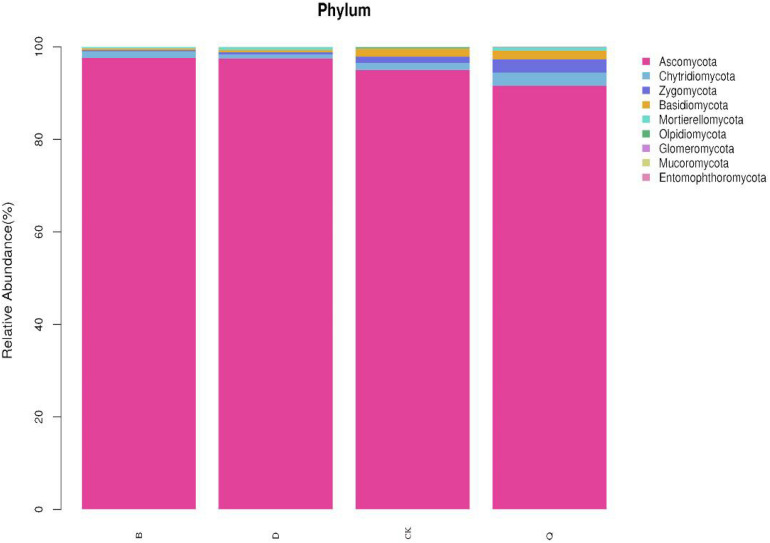
Relative abundance of dominant fungi in continuous tomato cropping substrates under different treatments. Fungal phylum in different rotation treatments. Treatment abbreviations are defined in Figure 1.

There were differences in fungal community composition among treatments. At the phylum level, the dominant fungal phyla for treatments B and D were *Ascomycota*, with 97.62 and 97.47% relative abundance, respectively, and *Chytridiomycota*, with 1.41 and 0.91% relative abundance, respectively (Figure 2). The dominant fungal phyla in treatments Q and control CK were *Ascomycota*, *Chytridiomycota*, *Zygomycota*, and *Basidiomycota*, with relative abundances of 95.00, 1.55, 1.34, and 1.69%, respectively, in treatment Q, and 91.58, 2.87, 2.86, and 1.89%, respectively, in control CK. Compared with control CK, *Ascomycota* relative abundance in treatments B and D increased by 2.76 and 2.6%, respectively, but it decreased by 3.6% in treatment Q. Compared with control CK, *Chytridiomycota*, *Zygomycota*, and *Basidiomycota* relative abundances decreased by 9.03, 82.09, 78.11%, respectively, in treatment B, and by 41.29, 64.93, and 72.19%, respectively, in treatment D. Contrastingly, *Chytridiomycota*, *Zygomycota*, and *Basidiomycota* in treatment Q increased by 85.16, 113.43, and 11.83%, respectively, compared with control CK.

**FIGURE 3:**
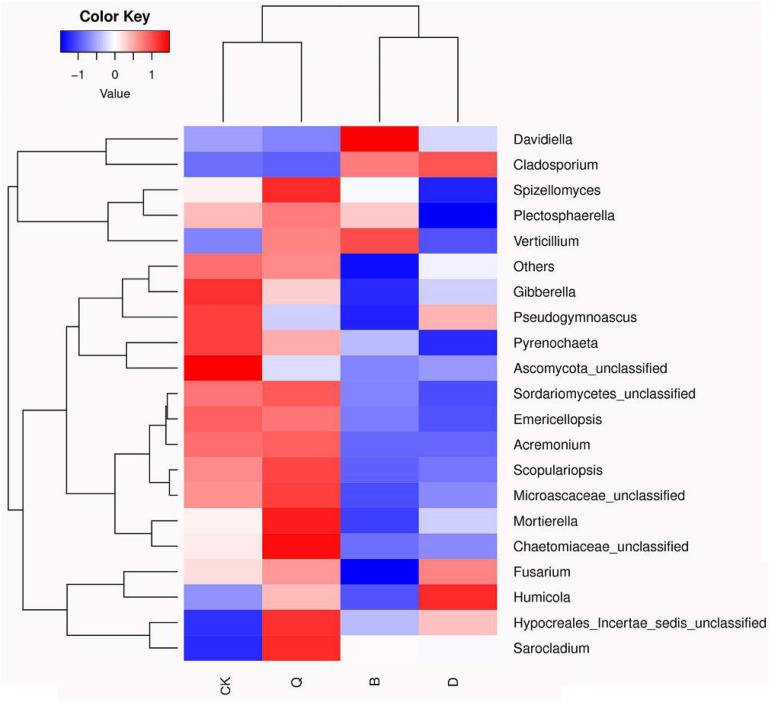
Heatmap analysis of fungal communities in continuous tomato cropping substrates under different treatments. The color of relative abundance of the community changed from blue to red, indicating that the relative abundance of the community changed from low to high. Treatment abbreviations are defined in Figure 1.

The fungal community relative abundance was further identified by heatmap analysis. According to the similarity comparison, the colonial structure of fungi in all treatments was divided into four groups (Figure 3). Treatments B and D were clustered into one group, and Q and CK were clustered into the other, indicating that there were significant differences in the community structure among the communities. The abundances of *Gibberella*, *Pseudogymnoascus*, *Pyrenochaeta*, and *Ascomycota_unclassified* were significantly higher in control CK than in treatment B, D, and Q. However, in treatment B, *Davidiella*, *Cladosporium*, and *Verticillium* abundances were higher than those in control CK. In treatment D, *Cladosporium* and *Humicola* abundances were higher than those in control CK.

As shown in Table 3, 19 fungi with significant difference between continuous cropping of tomato and continuous cropping of cabbage, bean and celery were selected for *t*-test (*P* < 0.05) (Supplementary Tables S1, S2). The abundance of *Cladosporium*, *Verticillium*, *Sarocladium*, *Davidella*, and *Hypocraeles-incertae-sedis-unclassified* was significantly higher than CK, while that of *Pseudogymnoascus*, *Microascaea-unclassified*, *Gibberella*, *Chaetomiaceae-unclassified*, A*scomycota-unclassified*, *Pyrenochaeta*, *Acremonium*, *Fusarium, Emericellopsis*, *Sordariomycetes*, and *Scopulariopsis* was significantly lower than CK. In D treatment, the abundance of *Cladosporium*, *Humiola*, and *Hypocraeles-incertae-sedis-unclassified* was significantly higher than CK, and the abundance of *Plectosaphaerella*, *Pseudonymnosacus*, *Microascaea-unclassified*, *Gibberella*, *Ascomycota-unclassified*, *Pyrenochaeta*, *Emericellopsis*, *Sordariomycetes-unclassified*, and *Scoreriopsis* was significantly lower than CK. In Q treatment, the abundance of *Mortierella*, *Humiola*, *Sarocladium*, and *Hypocraeles-incertae-sedis-unclassified* was significantly higher than CK, while that of *Pseudonymnoascus*, *Gibberella*, *Ascomycota-unclassified*, and *Pyrenochaeta* was significantly lower than CK.

**TABLE 3:** Comparison of fungal genus in different vegetable rotations and continuous tomato cropping substrates (*t*-test).

**Genus**	**log_2_FC**	***p*-value**
	**B/CK**	(*p < 0.05)
*Cladosporium*	1.17	0.0495*
*Pseudogymnoascus*	–7.36	0.0495*
*Verticillium*	5.52	0.0495*
*Microascaceae_unclassified*	–1.28	0.0495*
*Gibberella*	–2.05	0.0495*
*Chaetomiaceae_unclassified*	–0.64	0.0495*
*Ascomycota_unclassified*	–3.36	0.0495*
*Pyrenochaeta*	–2.58	0.0495*
*Acremonium*	–0.74	0.0495*
*Sarocladium*	1.09	0.0495*
*Fusarium*	–2.21	0.0495*
*Davidiella*	0.88	0.0495*
*Emericellopsis*	–1.31	0.0495*
*Sordariomycetes_unclassified*	–1.84	0.0495*
*Scopulariopsis*	–2.09	0.0495*
*Hypocreales_Incertae_sedis_unclassified*	1.36	0.0495*

**Genus**	**log_2_FC**	***p*-value**

	**D/CK**	(*p < 0.05)
*Cladosporium*	1.34	0.0495*
*Plectosphaerella*	–3.08	0.0495*
*Pseudogymnoascus*	–2.01	0.0495*
*Microascaceae_unclassified*	–1.02	0.0495*
*Gibberella*	–1.24	0.0495*
*Ascomycota_unclassified*	–3.16	0.0495*
*Humicola*	5.55	0.0495*
*Pyrenochaeta*	–4.04	0.0495*
*Emericellopsis*	–1.48	0.0495*
*Sordariomycetes_unclassified*	–2.27	0.0495*
*Scopulariopsis*	–1.94	0.0495*
*Hypocreales_Incertae_sedis_unclassified*	2.61	0.0495*

**Genus**	**log2FC**	***p*-value**

	**Q/CK**	(*p < 0.05)
*Pseudogymnoascus*	–4.33	0.0495*
*Gibberella*	–0.77	0.0495*
*Ascomycota_unclassified*	–2.57	0.0495*
*Mortierella*	1.34	0.0495*
*Humicola*	3.12	0.0495*
*Pyrenochaeta*	–1.08	0.0495*
*Sarocladium*	2.19	0.0495*
*Hypocreales_Incertae_sedis_unclassified*	4	0.0495*

*Significant at the 0.05 probability level. B, D, Q, and CK are defined in Figure 1. FC in log_2_ FC is fold change, which represents the ratio of the expression amount between two samples (groups). After taking the logarithm based on 2, it is log_2_ FC.

### Effects of Environmental Factors on Fungal Community Distribution in Continuous Tomato Cropping Substrate

**FIGURE 4:**
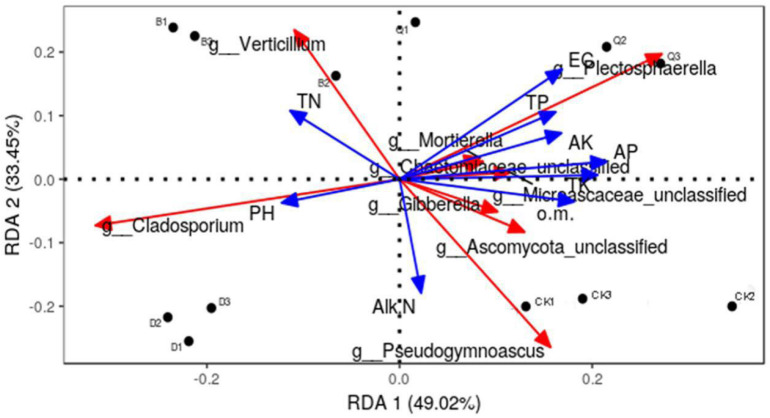
Effect of environmental factors on the distribution of fungal communities in continuous tomato cropping substrates. Dots represent matrix samples; blue arrows represent the physical and chemical properties of the matrix; and red arrows represent the matrix microorganisms. The angle between the influencing factors (between the factors and the microorganisms) indicate a positive correlation between two factors (acute angle) and a negative correlation (obtuse angle), and the arrow length represents the strength of the effect for each factor. EC, matrix conductivity; pH, matrix pH; o.m., matrix organic matter; TN, total nitrogen; TP, total phosphorus; TK, total potassium; AP, available phosphorus; AK, available potassium; Alk N, alkali nitrogen. Treatment abbreviations are defined in Figure 1.

RDA was used to analyze the relationship between the structure (relative abundance) of 10 fungal communities and the physicochemical properties of their substrate matrices (Figure 4). The fungal community in each treatment matrix was classified by two axes, which explained 82.47% of the total variability. Abundant fungal genera in control CK were positively correlated with organic matter, alkali-soluble N, and total K and negatively correlated with total N and pH. Contrastingly, those in treatment Q were positively correlated with EC, total P, available P, and available K, while those in treatment B were positively correlated with total N, and those in D treatment were positively correlated with total N and pH.

## Discussion

With increasing years of continuous cropping, the soil organic matter, total N, available P, available K, and alkali-soluble N contents all showed a continuous downward trend (Li et al., 2017), while crop rotation or intercropping can effectively alleviate the soil nutrient decline and imbalance caused by single crop continuous cropping (Latif et al., 1992; Long et al., 1999). Our results showed that substrate EC, organic matter, total K, and available P were significantly higher after rotation with cabbage and kidney bean than in continuous tomato cropping (Table 1), which indicated that the rotation of Chinese cabbage or bean could change the physical and chemical properties of the substrate of continuous cropping of tomato and significantly improve the nutrient supply of the substrate of continuous cropping of tomato. In the study of Costa and Crusciol (2016), it was also proven that rotation could increase the input of organic carbon in soil. But the substrate pH and alkeline-N of rotation celery were significantly higher than that of tomato continuous cropping (Table 1). Some researchers indicated that the proportion of fungi in the soil would decrease with the decrease in soil pH in boreal forest acid soil (Högberg et al., 2003) or alkaline soil (Alfaro et al., 2017), which indicated that rotation celery could increase the proportion of fungi in the continuous cropping substrate.

Crop continuous cropping can alter the structure of fungal community structure in soil (Xiong et al., 2015; Liu et al., 2019). In our research results, the PCoA of the matrix fungal community structure of the continuous tomato cropping that was subjected to vegetable rotation identified a clear separation of the communities (Figure 1B), which was also confirmed by the hierarchical clustering (Figure 1A). That is to say, the use of different cultivation techniques can also cause significant differences in fungal genetic communities in the soil, leading to characteristic changes in fungal community structure (Lupwayi et al., 1998; Brussaard et al., 2007; Ghimire et al., 2014). Rotation of different vegetables can have different effects on the richness and diversity of fungi in continuous cropping matrix. Rotation of celery could improve the richness and diversity of fungi in continuous cropping matrix, while rotation of cabbage and bean can reduce the richness and diversity of fungi in continuous cropping matrix (Table 2). Celery is a kind of vegetable with strong allelochemicals. Rotation with crops with stronger allelochemicals can effectively improve the richness and diversity of soil fungi (Ding et al., 2018). It has also been shown that continuous cropping can increase the fungal richness of crop rhizosphere soil (Meng et al., 2012; Zhou and Wu, 2012). Rotation can effectively reduce the amount of culturable fungi in soil (Wei et al., 2012). In our study, rotation cabbage and bean treatment reduced the richness and diversity of fungi in tomato continuous cropping matrix (Table 2). This may due to the different root exudates of rotation soil, which can reduce the self-toxicity of tomatoes, inhibit the propagation of pathogenic bacteria, and improve the microecological environment of soil (Alvey et al., 2003; Zhu and Fox, 2003). It is an important discovery that rotation of different vegetables will have opposite effect on the diversity and richness of fungi in continuous cropping matrix.

Fungi can decompose soil organic matter and play an important role in the terrestrial ecosystem (Abed et al., 2013; Peay et al., 2013; Acosta-Martínez et al., 2014). Among them, *Chytridiomycota*, *Zygomycota*, and *Basidiomycota* are the main media of organic matter decomposition in most terrestrial ecosystems (James et al., 2006; Richardson, 2009). Compared with tomato continuous cropping (CK), rotation celery (Q) increased the relative abundance of *Chytridiomycota*, *Zygomycota*, and *Basidiomycota* (Figure 2). Qin et al. (2017) also found that the relative abundance of *Zygomycota* and *Basidiomycota* in potato continuous cropping soil under ridge and furrow mulching cultivation mode was higher than that under flat ridge mulching cultivation mode. This indicated that rotation celery could increase the relative abundance of beneficial fungi in tomato continuous cropping substrates.

In addition, as shown in the fungal community heatmap, *Gibberella* and *Pyrenochaeta* abundances in cabbage, kidney bean, and celery rotations were significantly reduced compared with those of continuous tomato cropping (Figure 3). *Gibberella* is a well-known pathogen associated with diseases of several crops including rice, sugar cane, and corn (Marasas et al., 2006; Mohd et al., 2008; Hsuan et al., 2011). It has been reported that sugarcane–soybean intercropping can also reduce the relative abundance of *Gibberella* in soil (Lian et al., 2018). Furthermore, *Pyrenochaetalycopersici* is a soil-borne fungus that causes root rot of tomato wood. After infection with pathogens, tomato roots form lesions and wrinkles, and finally form a cork structure (Jones et al., 1989). With increasing continuous tomato planting time, pathogens accumulate in the soil and the incidence of *Pyrenochaetalycopersici* is multiplied (Last et al., 2010). The use of biological control, such as treatment of tomato plants with *Trichodermaviride* 18/17 SS, *Streptomyces* spp. AtB42, and *Bacillus subtilis* M51 PI, can effectively prevent the growth of *Pyrenochaetalycopersici* (Fiume and Fiume, 2008). The rotation method used in the present study alleviated the soil-borne disease caused by continuous tomato cropping. We found that rotation of cabbage, kidney beans, and celery can significantly reduce *Pyrenochaeta* abundance in continuous tomato cropping substrate (Table 3).

Rotation is a good practice to protect vegetable production. By changing the soil microenvironment, it can effectively improve soil physical and chemical properties, regulate soil fertility, and increase crop yield (Jahan et al., 2016; Lahouar et al., 2016). Several studies have shown that environmental factors shape fungal communities and structures (Liu et al., 2015; Lahouar et al., 2016). In the present study, we used RDA plots to show the interaction between physicochemical properties of the substrate and 10 fungal communities of interest. The most abundant taxa were found to be strongly negatively correlated with matrix pH and total N (Figure 4). Our findings are consistent with other studies, showing that soil pH is a very important factor for building microbial communities (Rousk et al., 2010; Jun et al., 2014; Ding et al., 2018). The EC value of substrate not only affects the growth of rhizosphere, but also affects the development of fungi. Our study shows that the EC of the substrate after planting celery is 413.33 μS/cm (Table 1), and the study of Li et al. (2010) also showed that celery can grow normally within the EC Value of 200–400 μS/cm. In our study, the EC of substrate was negatively correlated with the richest taxa (Figure 4), which is consistent with previous research results (Rousk et al., 2010; Zhao et al., 2014; Ding et al., 2018). In contrast, the most abundant taxa were positively correlated with the matrices total P, total K, available P, available K, organic matter, and EC (Figure 4). Our findings are consistent with previous studies showing that soil physicochemical properties have a strong impact on soil microbial communities and structures (Rousk et al., 2010; Fernández-Calviño and Bååth, 2016; Hietala et al., 2016).

## Conclusion

In this experiment, the effects of Chinese cabbage, kidney bean, and celery rotation on the physical and chemical properties, fungal diversity, and community structure of continuous tomato cropping rhizosphere were studied. Rotating different vegetables improved the physical and chemical properties of the substrate under greenhouse continuous tomato cropping. Rotating cabbage and kidney bean reduced the diversity and abundance of fungi, which changed the fungal community structure of the substrate, while celery rotation increased fungal diversity and abundance of continuous cropping and also reduced the relative abundance of harmful fungal genera. Therefore, from the perspective of maintaining the balance of the substrate microbial ecological environment, we believe that the rotation of celery can better solve the problems arising from continuous tomato cropping.

## Data Availability Statement

The datasets (SRP253823) for this study can be found in the NCBI Sequence Read Archive (https://identifiers.org/ncbi/insdc.sra:SRP253823).

## Author Contributions

JY and JL designed the work. LJ, NJ, and LN performed the work. LJ analyzed the data. JX, XX, LH, ZT, and YW revised the manuscript. All authors approved the final version of this manuscript.

## Conflict of Interest

The authors declare that the research was conducted in the absence of any commercial or financial relationships that could be construed as a potential conflict of interest.

## References

[B1] Abed R. M. M. Al-Sadi A. M. Al-Shehi M. Al-Hinai S. Robinson M. D. (2013). Diversity of free-living and lichenized fungal communities in biological soil crusts of the Sultanate of Oman and their role in improving soil properties. *Soil Biol. Biochem.* 57 695–705. 10.1016/j.soilbio.2012.07.023

[B2] Acosta-Martínez V. Cotton J. Gardner T. Moore-Kucera J. Zak J. Wester D. (2014). Predominant bacterial and fungal assemblages in agricultural soils during a record drought/heat wave and linkages to enzyme activities of biogeochemical cycling. *Appl. Soil Ecol.* 84 69–82. 10.1016/j.apsoil.2014.06.005

[B3] Alfaro F. D. Manzano M. Marquet P. A. Gaxiola A. (2017). Microbial communities in soil chronosequences with distinct parent material: the effect of soil pH and litter quality. *J. Ecol.* 105 1709–1722. 10.1111/1365-2745.12766

[B4] Alvey S. Yang C. H. Bürkert A. Crowley D. E. (2003). Cereal/legume rotation effects on rhizosphere bacterial community structure in West African soils. *Biol. Ferti. Soils* 37 73–82. 10.1007/s00374-002-0573-2

[B5] Bao S. (2000). *Soil Agrochemical Analyses*, 3rd Edn Beijing: China Agriculture Press.

[B6] Baumann D. T. Bastiaans L. Kropff M. J. (2001). Effects of intercropping on growth and reproductive capacity of late-emerging *Senecio vulgaris* L., with special reference to competition for light. *Ann. Bot.* 87 209–217. 10.1006/anbo.2000.1320 32050737

[B7] Baumann D. T. Kropff M. J. Bastiaans L. (2000). Intercropping leeks to suppress weeds. *Weed Res.* 40 359–374. 10.1046/j.1365-3180.2000.00197.x

[B8] Bokulich N. A. Subramanian S. Faith J. J. Gevers D. Gordon J. I. Knight R. (2013). Quality-filtering vastly improves diversity estimates from Illumina amplicon sequencing. *Nat. Methods* 10 57–59. 10.1038/nmeth.2276 23202435 PMC3531572

[B9] Brussaard L. Ruiter P. C. D. Brown G. G. (2007). Soil biodiversity for agricultural sustainability. *Agricul. Ecosyst. Environ.* 121 233–244.

[B10] Caporaso J. G. Kuczynski J. Stombaugh J. Bittinger K. Bushman F. D. Costello E. K. (2010). QIIME allows analysis of high-throughput community sequencing data. *Nat. Methods.* 7 335–336. 10.1038/nmeth.f.303 20383131 PMC3156573

[B11] Cole J. R. Wang Q. Cardenas E. Fish J. Chai B. Farris R. J. (2009). The ribosomal database project: improved alignments and new tools for rRNA analysis. *Nucleic Acids Res.* 37 D141–D145. 10.1093/nar/gkn879 19004872 PMC2686447

[B12] Cole J. R. Wang Q. Fish J. A. Chai B. McGarrell D. M. Sun Y. (2014). Ribosomal database project: data and tools for high throughput rRNA analysis. *Nucleic Acids Res.* 42 D633–D642. 10.1093/nar/gkt1244 24288368 PMC3965039

[B13] Costa C. H. M. D. Crusciol C. A. C. (2016). Long-term effects of lime and phosphogypsum application on tropical no-till soybean–oat–sorghum rotation and soil chemical properties. *Eur. J. Agron.* 74 119–132. 10.1016/j.eja.2015.12.001

[B14] Ding H. Ali A. Cheng Z. (2018). Dynamics of a soil fungal community in a three-year green garlic/cucumber crop rotation system in Northwest China. *Sustainability* 10:1391 10.3390/su10051391

[B15] Fernández-Calviño D. Bååth E. (2016). Interaction between pH and Cu toxicity on fungal and bacterial performance in soil. *Soil Biol. Biochemi.* 96 20–29.

[B16] Fiume G. Fiume F. (2008). Biological control of corky root in tomato. *Commun. Agric. Appl. Biol. Sci.* 73 233–248. 19226760

[B17] Fu H. Zhang G. Zhang F. Sun Z. Geng G. Li T. (2017). Effects of continuous tomato monoculture on soil microbial properties and enzyme activities in a solar greenhouse. *Sustainability* 9:317 10.3390/su9020317

[B18] Garbeva P. van Veen J. A. van Elsas J. D. (2004). Microbialdiversityinsoil: selection of microbial populations by plant and soil type and implications for disease suppressiveness. *Annu. Rev. Phytopathol.* 42 243–270. 10.1146/annurev.phyto.42.012604.135455 15283667

[B19] Ghimire R. Norton J. B. Stahl P. D. Norton U. (2014). Soil microbial substrate properties and microbial community responses under irrigated organic and reduced-tillage crop and forage production systems. *PLoS One* 8:e0103901 10.1371/journal.pone.0103901 PMC4121199 25090235

[B20] Han L. L. Wang J. T. Yang S. H. Chen W. F. Zhang L. M. He J. Z. (2016). Temporal dynamics of fungal communities in soybean rhizosphere. *J. Soils Sediments* 17 1–8. 10.1007/s11368-016-1534-y

[B21] Hietala A. M. Nagy N. E. Burchardt E. C. Solheim H. (2016). Interactions between soil pH, wood heavy metal content and fungal decay at Norway spruce stands. *Appl. Soil Ecol.* 107 237–243. 10.1016/j.apsoil.2016.06.008

[B22] Högberg M. N. Bååth E. Nordgren A. Arnebrant K. Högberg P. (2003). Contrasting effects of nitrogen availability on plant carbon supply to mycorrhizal fungi and saprotrophs —a hypothesis based on fifield observations in boreal forest. *New Phytol.* 160 225–238. 10.1046/j.1469-8137.2003.00867.x 33873541

[B23] Hsuan H. M. Salleh B. Zakaria L. (2011). Molecular identification of fusarium species in Gibberellafujikuroispecies complex from rice, sugarcane and maize from peninsular malaysia. *Int. J. Mol. Sci.* 12 6722–6732. 10.3390/ijms12106722 22072914 PMC3211005

[B24] Jahan M. A. H. S. Hossain A. Sarkar M. A. R. Silva J. A. T. D. Ferdousi M. N. S. (2016). Productivity impacts and nutrient balances of an intensive potato-mungbean-rice crop rotation in multiple environments of Bangladesh. *Agric. Ecosyst. Environ.* 231 79–97. 10.1016/j.agee.2016.06.032

[B25] James T. Y. Letcher P. M. Longcore J. E. Mozley-Standridge S. E. Porter D. Powell M. J. (2006). A molecular phylogeny of the flagellated fungi (*Chytridiomycota*) and description of a new phylum (*Blastocladiomycota*). *Mycologia* 98 860–871. 10.1080/15572536.2006.11832616 17486963

[B26] Jing Q. Y. (1999). Allelopathic suppression of *Pseudomonas* solanacearum infection of tomato (*Lycopersiconesculentum*) in a tomato-chinese chive (*Allium tuberosum*) intercropping system. *J. Chem. Ecol.* 25 2409–2417. 10.1023/a:1020809805081

[B27] Jones R. A. Millett A. Giannini C. (1989). A greenhouse screening method for *Pyrenochaeta lycopersicire* sistance in tomatoes. *Euphytica* 40 187–191. 10.1007/bf00024510

[B28] Jun Z. Tian N. Yong L. Wu X. Wei R. Biao S. (2014). Responses of bacterial communities in arable soils in a rice-wheat cropping system to different fertilizer regimes and sampling times. *PLoS One* 9:e85301 10.1371/journal.pone.0085301 PMC3896389 24465530

[B29] Lahouar A. Marin S. Crespo-Sempere A. Saïda S. Sanchis V. (2016). Effects of temperature, water activity and incubation time on fungal growth and aflatoxin B1 production by toxinogenic *Aspergillus flavus* isolates on sorghum seeds. *Rev. Argent. Microbiol.* 48:S0325754115001297 10.1016/j.ram.2015.10.001 26920121

[B30] Last F. T. Ebben M. H. Hoare R. C. Turner E. A. Carter A. R. (2010). Build-up of tomato brown root rot caused by Pyrenochaetalycopersici Schneider and Gerlach. *Ann. Appl. Biol.* 64 449–459. 10.1111/j.1744-7348.1969.tb02894.x

[B31] Latif M. A. Mehuys G. R. Mackenzie A. F. Alli I. Faris M. A. (1992). Effects of legumes on soil physical quality in a maize crop. *Plant Soil* 140 15–23. 10.1007/bf00012802

[B32] Lee S. Yeo I. Y. Sadeghi A. M. Mccarty G. W. Hively W. D. Lang M. W. (2016). Impacts of watershed characteristics and crop rotations on winter cover crop nitrate-nitrogen uptake capacity within agricultural watersheds in the chesapeakebay region. *PLoS One* 11:e0157637 10.1371/journal.pone.0157637 PMC4924834 27352119

[B33] Li J. G. Ren G. D. Jia Z. J. Dong Y. H. (2014). Composition and activity of rhizosphere microbial communities associated with healthy and diseased greenhouse tomatoes. *Plant Soil* 380 337–347. 10.1007/s11104-014-2097-6

[B34] Li T. Liu T. Zheng C. Kang C. Yang Z. Yao X. (2017). Changes in soil bacterial community structure as a result of incorporation of Brassica plants compared with continuous planting eggplant and chemical disinfection in greenhouses. *PLoS One* 12:e0173923 10.1371/journal.pone.0173923 PMC5367839 28346463

[B35] Li Y. Wang T. Li J. Ao Y. (2010). Effect of phosphorus on celery growth and nutrient uptake under different calcium and magnesium levels in substrate culture. *Hortic. Sci.* 37 99–108. 10.17221/81/2009-HORTSCI

[B36] Lian T. Mu Y. Ma Q. Cheng Y. Gao R. Cai Z. (2018). Use of sugarcane–soybean intercropping in acid soil impacts the structure of the soil fungal community. *Sci. Rep.* 8:14488 10.1038/s41598-018-32920-2 PMC6160455 30262899

[B37] Liang M. Q. Zhang C.-F. Peng C.-L. Lai Z.-L. Chen D.-F. Chen Z.-H. (2011). Plant growth, community structure, and nutrient removal in monoculture and mixed constructed wetlands. *Ecolo. Eng.* 37 309–316. 10.1016/j.ecoleng.2010.11.018

[B38] Liu H. Pan F. Han X. Song F. Zhang Z. Yan J. (2019). Response of soil fungal community structure to long-term continuous soybean cropping. *Front. Microbiol.* 9:3316 10.3389/fmicb.2018.03316 PMC6333693 30687292

[B39] Liu J. Sui Y. Yu Z. Shi Y. Chu H. Jin J. (2015). Soil carbon content drives the biogeographical distribution of fungal communities in the black soil zone of northeast China. *Soil Biol. Biochem.* 83 29–39. 10.1016/j.soilbio.2015.01.009

[B40] Liu T. Cheng Z. Meng H. Ahmad I. Zhao H. (2014). Growth, yield and quality of spring tomato and physicochemical properties of medium in a tomato/garlic intercropping system under plastic tunnel organic medium cultivation. *Sci. Hortic.* 170 159–168. 10.1016/j.scienta.2014.02.039

[B41] Liu W. K. Yang Q. C. Du L. F. (2009). Soilless cultivation for high-quality vegetables with biogas manure in China: feasibility and benefit analysis. *Ren. Agric. Food Syst.* 24 300–307. 10.1017/S1742170509990081

[B42] Long L. Yang S. Li X. Zhang F. Christie P. (1999). Interspecific complementary and competitive interactions between intercropped maize and faba bean. *Plant Soil* 212 105–114. 10.1023/a:1004656205144

[B43] Lozupone C. Hamady M. Knight R. (2006). Unifrac - an online tool for comparing microbial community diversity in a phylogenetic context. *BMC Bioinformatics* 7:371 10.1186/1471-2105-7-371 PMC1564154 16893466

[B44] Lu L. Yin S. Liu X. Zhang W. Gu T. Shen Q. (2013). Fungal networks in yield-invigorating and-debilitating soils induced by prolonged potato monoculture. *Soil Biol. Biochem.* 65 186–194. 10.1016/j.soilbio.2013.05.025

[B45] Lupwayi N. Z. Rice W. A. Clayton G. W. (1998). Soil microbial diversity and community structure under wheat as influenced by tillage and crop rotation. *Soil Biol. Biochemi.* 30 1733–1741. 10.1016/s0038-0717(98)00025-x

[B46] Maboko M. M. Bertling I. Plooy C. P. D. (2013). Arbuscular mycorrhiza has limited effects on yield and quality of tomatoes grown under soilless cultivation. *Acta Agric. Scand.* 63 261–270. 10.1080/09064710.2012.755219

[B47] Marasas W. F. O. Ploetz R. C. Wingfield M. J. Wingfield B. D. Steenkamp E. T. (2006). Mango malformation disease and the associated Fusarium species. *Phytopathology* 96 667–672. 10.1094/PHYTO-96-0667 18943188

[B48] Meng P. P. Liu X. Qiu H. Z. Zhang W. M. Zhang C. H. Wang D. (2012). Fungal population structure and its biological effect in rhizosphere soil of continuously cropped potato. *YingyongShengtaiXuebao* 23:3079 10.1111/j.1949-8594.1906.tb02960.x 23431794

[B49] Mohd Z. I. N. Razak A. Salleh B. (2008). Bakanaedisease of rice in malaysia and indonesia: etiology of the causal agent based on morphological, physiological and pathogenicity characteristics. *J. Plant Prot. Res.* 48 475–485. 10.2478/v10045-008-0056-z

[B50] Nilsson R. H. Larsson K. H. Taylor A. F. S. Bengtsson-Palme J. Jeppesen T. S. Schigel D. (2019). The UNITE database for molecular identification of fungi: handling dark taxa and parallel taxonomic classifications. *Nucleic Acids Res.* 47 D259–D264. 10.1093/nar/gky1022 30371820 PMC6324048

[B51] Oksanen F. Blanchet F. G. Kindt R. Legendre P. Minchin P. R. O. Hara R. B. (2016). *Vegan**: Community Ecology Package. R Package Version 2.3-5*. Available online at: https://cran.r-project.org (accessed April 16, 2019).

[B52] Peay K. G. Baraloto C. Fine P. V. A. (2013). Strong coupling of plant and fungal community structure across western Amazonian rainforests. *ISME J.* 7 1852–1861. 10.1038/ismej.2013.66 23598789 PMC3749505

[B53] Penton C. R. Gupta V. V. Tiedje J. M. Neate S. M. Ophel-Keller K. Gillings M. (2014). Fungal community structure in disease suppressive soils assessed by 28S LSU gene sequencing. *PLoS One* 9:e93893 10.1371/journal.pone.0093893 PMC3974846 24699870

[B54] Qin S. Yeboah S. Xu X. Li Y. Yu B. (2017). Analysis on fungal diversity in rhizosphere soil of continuous cropping potato subjected to different furrow-ridge mulching managements. *Front. Microbiol.* 8:845 10.3389/fmicb.2017.00845 PMC5423957 28539923

[B55] R Core Team (2017). *R: A Language and Environment for Statistical Computing.* Vienna: R. (Foundation) for Statistical Computing.

[B56] Richardson M. (2009). The ecology of the Zygomycetes and its impact on environmental exposure. *Clin. Microbiol. Infect* 15 (Suppl. 5), 2–9. 10.1111/j.1469-0691.2009.02972 19754749

[B57] Rodrigo M. Marco K. Irene D. B. Ester D. Menno V. D. V. Schneider J. H. M. (2011). Deciphering the rhizosphere microbiome for disease-suppressive bacteria. *Science* 332 1097–1100. 10.1126/science.1203980 21551032

[B58] Rognes T. Flouri T. Nichols B. Quince C. Mahe F. (2016). VSEARCH: a versatile open source tool for metagenomics. *PeerJ* 4:e2584 10.7717/peerj.2584 PMC5075697 27781170

[B59] Rousk J. Baath E. Brookes P. C. Lauber C. L. Lozupone C. Caporaso J. G. (2010). Soil bacterial and fungal communities across a pH gradient in an arable soil. *Isme J.* 4 1340–1351. 10.1038/ismej.2010.58 20445636

[B60] Sommermann L. Geistlinger J. Wibberg D. Deubel A. Zwanzig J. Babin D. (2018). Fungal community profiles in agricultural soils of a long-term field trial under different tillage, fertilization and crop rotation conditions analyzed by high-throughput ITS-amplicon sequencing. *PLoS One* 13:0195345 10.1371/journal.pone.0195345 PMC5886558 29621291

[B61] Tago K. Itoh H. Kikuchi Y. Hori T. Hayatsu M. (2014). A fine-scale phylogenetic analysis of free-living Burkholderiaspecies in sugarcane field soil. *Microbes Environ.* 29 434–437. 10.1264/jsme2.ME14122 25410730 PMC4262370

[B62] Tao R. Hui L. Lu J. Bu R. Li X. Cong R. (2015). Crop rotation-dependent yield responses to fertilization in winter oilseed rape(Brassica napus L.). *Crop J.* 3 396–404. 10.1016/j.cj.2015.04.007

[B63] Tian Y. Zhang X. Liu J. Chen Q. Gao L. (2009). Microbial properties of rhizosphere soils as affected by rotation, grafting, and soil sterilization in intensive vegetable production systems. *Sci. Hortic.* 123 139–147. 10.1016/j.scienta.2009.08.010

[B64] Tian Y. Zhang X. Liu J. Gao L. (2011). Effects of summer cover crop and residue management on cucumber growth in intensive Chinese production systems: soil nutrients, microbial properties and nematodes. *Plant Soil* 339 299–315. 10.1007/s11104-010-0579-8

[B65] Van Assche C. Vangheel M. (1993). “Special phytopathological problems in soilless cultures and substrate cultures,” in *International Symposium on New Cultivation Systems in Greenhouse*, Vol. 361 (Leuven: ISHS), 355–360. 10.17660/ActaHortic.1994.361.35

[B66] Wei L. I. Cheng Z. H. Meng H. W. Jing Z. Jing L. Xue-Jiao L. (2012). Effect of rotating different vegetables on micro-biomass and enzyme in tomato continuous cropped substrate and afterculturetomato under plastic tunnel cultivation. *Acta Hortic. Sin.* 39 73–80. 10.16420/j.issn.0513-353x.2012.01.021

[B67] Weller D. M. Landa B. B. Mavrodi O. V. Schroeder K. L. Fuente L. De La Bankhead S. (2006). Role of 2,4-diacetylphloroglucinol-producing fluorescent *Pseudomonas* spp. in the defense of plant roots. *Plant Biol.* 9 4–20. 10.1055/s-2006-924473 17058178

[B68] Wright P. J. Falloon R. E. Hedderley D. (2015). Different vegetable crop rotations affect soil microbial communities and soilborne diseases of potato and onion: literature review and a long-term field evaluation. *N. Z. J. Crop Hortic. Sci.* 43 85–110. 10.1080/01140671.2014.979839

[B69] Xiong W. Zhao Q. Zhao J. Xun W. Li R. Zhang R. (2015). Different continuous cropping spans significantly affect microbial community membership and structure in a vanilla-grown soil as revealed bydeeppyrosequencing. *Microb. Ecol.* 70 209–218. 10.1007/s00248-014-0516-0 25391237

[B70] Zhang J. Kobert K. Flouri T. Stamatakis A. (2014). PEAR: a fast and accurate Illumina Paired-End reAd mergeR. *Bioinformatics* 30 614–620. 10.1093/bioinformatics/btt593 24142950 PMC3933873

[B71] Zhang Z. Y. Chen H. Yang Y. H. Chen T. Lin R. Y. Chen X. J. (2010). Effects of continuous cropping on bacterial community diversity in rhizosphere soil of Rehmanniaglutinosa. *Ying Yong Sheng Tai Xue Bao* 21 2843–2848. 10.3724/SP.J.1142.2010.40491 21361008

[B72] Zhao J. Zhang R. Xue C. Xun W. Sun L. Xu Y. (2014). Pyrosequencing reveals contrasting soil Bacterial diversity and community structure of two main winter wheat cropping systems in China. *Microb. Ecol.* 67 443–453. 10.1007/s00248-013-0322-0 24276539

[B73] Zhou X. Wu F. (2012). Dynamics of the diversity of fungal and Fusarium communities during continuous cropping of cucumber in the greenhouse. *Fems Microbiol. Ecol.* 80 469–478. 10.1111/j.1574-6941.2012.01312.x 22273443

[B74] Zhu Y. Fox R. H. (2003). Corn–soybean rotation effects on nitrate leaching. *Agron. J.* 95 1028–1033. 10.2134/agronj2003.1028

